# A comprehensive evaluation of potentially significant drug-drug, drug-herb, and drug-food interactions among cancer patients receiving anticancer drugs

**DOI:** 10.1186/s12885-022-09649-3

**Published:** 2022-05-14

**Authors:** Amer A. Koni, Maisa A. Nazzal, Bushra A. Suwan, Samah S. Sobuh, Najiya T. Abuhazeem, Asil N. Salman, Husam T. Salameh, Riad Amer, Sa’ed H. Zyoud

**Affiliations:** 1 Division of Clinical Pharmacy, Department of Hematology and Oncology, An-Najah National University Hospital, Nablus, 44839 Palestine; 2 Department of Clinical and Community Pharmacy, College of Medicine and Health Sciences, An-Najah National University, Nablus, 44839 Palestine; 3 Department of Clinical Pharmacy, An-Najah National University Hospital, Nablus, 44839 Palestine; 4 Department of Medicine, College of Medicine and Health Sciences, An-Najah National University, Nablus, 44839 Palestine; 5 Department of Hematology and Oncology, An-Najah National University Hospital, Nablus, 44839 Palestine; 6 Poison Control and Drug Information Center (PCDIC), College of Medicine and Health Sciences, An-Najah National University, Nablus, 44839 Palestine; 7 Clinical Research Center, An-Najah National University Hospital, Nablus, 44839 Palestine

**Keywords:** Drug-drug interactions, Drug-herb interactions, Drug-food interactions, Prevalence, Predictors, Palestine, Cancer, Anticancer drugs

## Abstract

**Introduction:**

During the cancer treatment path, cancer patients use numerous drugs, including anticancer, supportive, and other prescribed medications, along with herbs and certain products. This puts them at risk of significant drug interactions (DIs). This study describes DIs in cancer patients and their prevalence and predictors.

**Methods:**

A cross-sectional study design was used to achieve the study objectives. The study was carried out in two centers in the northern West Bank, Palestine. The Lexicomp® Drug Interactions tool (Lexi-Comp, Hudson OH, USA) was applied to check the potential DIs. In addition, the Statistical Package for the Social Sciences (SPSS) was used to show the results and find the associations.

**Results:**

The final analysis included 327 patients. Most of the participants were older than 50 years (61.2%), female (68.5%), and had a solid tumor (74.6%). The total number of potential DIs was 1753, including 1510 drug-drug interactions (DDIs), 24 drug-herb interactions, and 219 drug-food interactions. Importantly, the prevalence of DDIs was 88.1%. In multivariate analysis, the number of potential DDIs significantly decreased with the duration of treatment (*p* = 0.007), while it increased with the number of comorbidities (*p* < 0.001) and the number of drugs used (*p* < 0.001).

**Conclusions:**

We found a high prevalence of DIs among cancer patients. This required health care providers to develop a comprehensive protocol to monitor and evaluate DIs by improving doctor-pharmacist communication and supporting the role of clinical pharmacists.

## Introduction

Cancer is an expression used to describe a set of diseases characterized by uncontrolled cellular growth in any part of the body [1]. It poses a global health burden as its prevalence and death rate swiftly rise [1]. Recently, cancer has become the leading cause of death worldwide [1]. In 2020, 3191 new cancer cases were documented in Palestine, 32.8% being elderly (above 64 years) [2]. According to the last report by the Palestinian Ministry of Health in 2020, cancer was the third cause of death (14.1%) in Palestine [2].

Drug interaction (DI) occurs when the targeted drug is affected by another agent and results in alterations in its clinical effect [3]. This interaction may influence the pharmacokinetics, pharmacodynamics, or the targeted drug's pharmaceutical properties [4]. Consequently, it negatively impacts the patient’s quality of life by either potentiating the drug-related adverse effects or reducing its efficacy [5]. It is noteworthy that oncology patients are prone to such interactions for several reasons [5]. First, they are treated with more than one anticancer agent. Second, multiple drugs are prescribed to treat their chronic conditions [5]. Third, supportive medications are sometimes used to control therapy-related adverse effects or disease-related symptoms [5]. Fourth, they sometimes use alternative medicine (such as herbs) or over-the-counter (OTC) products [5]. In particular, 4% of cancer-related deaths are due to a severe drug-related event, including DIs [6].

Multiple articles were published from the developed and developing countries to describe the prevalence, predictors, and features of drug-drug interactions (DDIs) among cancer patients, which showed a variation in the prevalence of DDIs [7–17]. This could be due to differences in the prescribing pattern and the interaction tool. For example, in Palestine, two studies reported a remarkably high prevalence of DDIs among hemodialysis (89.1%) [18] and cardiovascular patients (94%) [19], while another study documented a prevalence of 56% in surgical patients [20].

Of note, DDIs among Palestinian oncology patients were not described previously. In addition, data regarding the interactions of drugs with herbs and other products were lacking. This makes research in this area paramount to identify and address potential DIs in the cancer population. Our study was designed to identify the prevalence of DIs among Palestinian oncology patients. Therefore, it will help formulate a clinical intervention method to minimize clinically significant DIs in this category. Furthermore, the study aimed to describe the main factors related to DDIs and determine their characteristics. And this work will be a fundamental reference for decision-makers to properly evaluate the profiles of the medications of patients and increase their awareness and knowledge of DIs.

## Methods

### Study design

A cross-sectional study design was used by including two main sources of information; cancer patients and medical records (electronic and patient files). Indeed, this work was done by interviewing all cancer patients who were attending the hospitals to receive their treatment between April 2021 and December 2021. The interviews were achieved by three clinical pharmacists who recorded all patients’ medications (including prescribed, OTC, and supportive care drugs) and miscellaneous products (including alcohol, caffeine, tobacco, and grapefruit) they take, and asked about the symptoms they complained at the time of interview. Moreover, detailed information about patients’ characteristics and anticancer treatment was extracted from medical records.

### Study setting

Our study took place in the oncology center at An-Najah National University Hospital and Al-Watani Hospital in Nablus, Palestine. These centers are considered the largest centers in Palestine and the main reference for pediatric and adult patients with hematologic and nonhematologic malignancies in Palestine; the West Bank and Gaza Strip.

### Study population and sampling procedure

According to medical records, the number of cancer patients visiting the two hospitals during the study period was around 2150. Using the Daniel formula [21]; *n* = Z2*P (1-P) / d2, where *n* = sample to be calculated when the population size is more than 10,000, z = 1.96 (CI of 95%), d = 0.05 (absolute precision as a margin of error), *P* = 0.5 expected prevalence or response distribution. The resulting sample size was 385 patients. As our population is less than 10,000 (*N* = 2,150), we adjusted this number using the adjusted sample equation; adjusted sample = n/ (1 + (n/N)). Therefore, the sample size was found to be 327 patients, with a 5% margin of error, a response of 50%, and a 95% confidence interval.

### Inclusion and exclusion criteria

Our study included all cancer patients aged 18 years or above with all cancer types and stages who came to the hospitals during the study period to take their anticancer medications in the outpatient setting. Patients who refused or were unable to participate, did not mention all their medications, had psychological problems, or were hospitalized were excluded.

### Data collection instrument

The data collection form contained variables regarding sociodemographic and clinical information of the patients, including age, sex, body mass index (BMI), education level, family history of cancer, type of cancer, the current state of disease, the intent of treatment, duration of treatment, current medications, use of miscellaneous agents, herbal use, and symptoms. Based on a previous study [7], all drugs used were classified into two subgroups, which are ≤ 7 drugs or > 7.

All medications, herbs, and other products of each patient were entered into a software program Lexicomp® Drug Interactions (Lexi-Comp, Hudson OH, USA), to assess the potential DIs and classify their clinical significance and implications. This program was chosen because it has high sensitivity and specificity and is sometimes used as a gold standard to compare DI tools [22, 23]. Previously, it was also used to evaluate the drug-dietary supplement interactions [24] and drug-herb interactions [25]. This instrument classifies DIs according to their risk rating (A = no known interaction, B = no action needed, C = monitor therapy, D = consider therapy modification, or X = avoid combination), severity (N/A, minor, moderate, or major), and reliability (poor, fair, good, or excellent). In addition, all potential DDIs were independently checked by three practicing clinical pharmacists to ensure data accuracy.

### Ethical approval

All aspects of the study protocol, including access to and use of the patient's clinical information, were approved by the Institutional Review Boards (IRB) and the local health authority. In addition, we fully explained all parts of the study to the patients and obtained their informed verbal consent before starting data collection.

### Statistical analysis

We used the Statistical Package for Social Sciences program (IBM-SPSS) version 21 to analyze the data. Frequencies and percentages were used to explain the results. In addition, the median and interquartile ranges were used to express the continuous variables. As appropriate, the Kruskal–Wallis test or Mann–Whitney U test was applied to check the differences between variables. The Spearman test was used to examine the correlation between continuous variables. Furthermore, multiple linear regression analysis was performed to predict the variables that had a significant correlation with the number of DDIs. A *p*-value of < 0.05 was highlighted as significant for all applied tests.

## Results

### Sociodemographic and clinical characteristics

Of the 375 patients, 23 refused or were unable to participate, 4 had a psychological problem, and 21 did not mention all their medications. Therefore, 327 cancer patients were included in the final study. 61.2% of our sample was above 50 years, 68.5% were female, and 36.1% were obese. Regarding cancer types, 25.4% had hematologic malignancies, whereas 74.6% had solid cancer (Table 1). The most frequent types were breast cancer (37.9%), colon cancer (12.2%), non-Hodgkin lymphoma (7.6%), and multiple myeloma (7.6%) (Table 2). Approximately 59% of the patients have been treated for less than a year. Approximately half of the respondents (49.5%) had chronic diseases other than their primary disease (cancer). 51.7% used more than seven medications and 38.8% were prescribed more than two anticancer drugs. The prevalence of herbal use was 56.0%. Furthermore, 81.0% and 29.7% of the patients regularly consumed caffeine and grapefruit, respectively (Table 1).

**Table 1:** Sociodemographic and clinical characteristics (*N* = 327)

Characteristic	Frequency (%)
**Age**	
≤ 50	127 (38.8%)
> 50	200 (61.2%)
**Gender**	
Male	103 (31.5%)
Female	224 (68.5%)
**BMI**	
< 18.5 (underweight)	7 (2.1%)
18.5–24.9 (healthy weight)	83 (25.4%)
25–29.9 (overweight)	119 (36.4%)
≥ 30 (obese)	118 (36.1%)
**Cancer type**	
Hematological	83 (25.4%)
Solid	244 (74.6%)
**Metastasis**	
No	195 (59.6%)
Yes	132 (40.4%)
**Palliative**	
No	200 (61.2%)
Yes	127 (38.8%)
**Duration of treatment (years)**	
< 1	193 (59.0%)
1–3	90 (27.5%)
> 3	44(13.5%)
**Number of comorbid diseases**	
0	165 (50.5)
1	91 (27.8)
≥ 2	71 (21.7)
**Family history**	
No	173 (52.9%)
Yes	154 (47.1%)
**All drugs used**	
≤ 7	158 (48.3%)
> 7	169 (51.7%)
**Anticancer therapy**	
≤ 2	200 (61.2%)
> 2	127 (38.8%)
**Education**	
Not educated	18 (5.5%)
Standard < 5	32 (9.8%)
Standard 5–10	105(32.1%)
Standard 11–12	100 (30.6%)
University qualification	72 (22.0%)
**Caffeine**	
No	62 (19.0%)
Yes	265 (81.0%)
**Tobacco**	
No	273 (83.5%)
Yes	54 (16.5%)
**Alcohol**	
No	324 (99.1%)
Yes	3 (0.9%)
**Grapefruit**	
No	230 (70.3%)
Yes	97 (29.7%)
**Herbs**	
No	144 (44.0%)
Yes	183 (56.0%)

**Table 2:** Distribution of the type of cancer in the study sample

Cancer type	Frequency (%)
Breast cancer	124 (37.9%)
Colon cancer	40 (12.2%)
Multiple myeloma (MM)	25 (7.6%)
Non-hodgkin lymphoma (NHL)	25 (7.6%)
Hodgkin lymphoma (HL)	21 (6.4%)
Lung cancer	21(6.4%)
Ovarian cancer	17 (5.2%)
Pancreatic cancer	12 (3.7%)
Prostate cancer	7 (2.1%)
Gastric cancer	5 (1.5%)
Acute myeloid leukemia (AML)	5 (1.5%)
Acute lymphoblastic leukemia (ALL)	3 (0.9%)
Uterine cancer	2 (0.6%)
Gallbladder cancer	2 (0.6%)
Leiomyosarcoma	2 (0.6%)
Chronic lymphocytic leukemia (CLL)	2 (0.6%)
Bladder cancer	1 (0.3%)
Ewing cancer	1 (0.3%)
Adenocarcinoma of the leg	1 (0.3%)
Neuroendocrine	1 (0.3%)
Osteosarcoma	1 (0.3%)
Other types	9 (2.8%)

### Reported symptoms

The number of symptoms reported among the study sample ranged from 0 and 16, with a mean ± SD of 6.8 ± 3.5. However, the most common symptoms reported by the respondents were as follows: generalized weakness (75.5%), body aches (66.4%), pallor (57.2%), anorexia (50.0%), and nausea and vomiting (47.4%) (Table 3).

**Table 3:** Reported symptoms at the time of the interview (*N* = 327)

Variable	Frequency (%)
Weakness	247 (75.5%)
Body aches	217 (66.4%)
Pallor	187 (57.2%)
Anorexia	180 (55.0%)
Nausea and vomiting	155 (47.4%)
Epigastric pain	137 (41.9%)
Abdominal distension	136 (41.6%)
Abdominal pain	126 (38.5%)
Sweating	123 (37.6%)
Swelling	90 (27.5%)
Dysphagia	84 (25.7%)
Shortness of breath	83 (25.4%)
Cough	80 (24.5%)
Urinary tract infection symptoms	79 (24.2%)
Loose motions	73 (22.3%)
Fever	70 (21.4%)
Constipation	67 (20.5%)
Bleeding	37 (11.3%)
Other symptoms	61 (18.7%)

### Description of DDIs

The total number of potential DIs was 1753, distributed as 1510 DDIs, 24 drug-herb interactions, and 219 drug-miscellaneous product interactions. Patients have used 1–19 drugs and the mean ± SD was 8.0 ± 3.2. The prevalence of DDIs among cancer patients was 88.1% (*N* = 288), 27.2% had 1–2 DDIs, 21.1% had 3–4 DDIs, and 12.8% had 5–6 DDIs, while 26.9% of the respondents had more than six DDIs. The characteristics of DDIs are presented in Table 4. Of all potential DDIs, 857 (56.8%) were classified as moderate interactions, while 13.2% were major interactions. In the risk rating classification, most of the DDIs (56.0%) had a ‘C’ category, 11.1% were ‘D’, and only 40 DDIs (2.6%) were considered ‘X’. Furthermore, the majority of DDIs had either fair (69.1%) or good (26.2%) reliability. The most frequent pairs of DDIs were Paracetamol/Ondansetron, Cyclophosphamide/Ondansetron, Doxorubicin/Cyclophosphamide, Metformin/Dexamethasone, and Dexamethasone/Aspirin (Table 5). However, combinations that should be avoided ‘X’ were Doxorubicin/Aprepitant (*n* = 20) and Promethazine/Metoclopramide (*n* = 6).

**Table 4:** Features of potential DDIs (1510)

No. of potential DDIs per subject (*N* = 327)	
No interactions	39 (11.9)
1–2	89 (27.2)
3–4	69 (21.1)
5–6	42 (12.8)
> 6	88 (26.9)
**Severity**	
Minor	424 (28.1)
Moderate	857 (56.8)
Major	199 (13.2)
N/A	30 (2.0)
**Risk rating**	
A	30 (2.0)
B	427 (28.3)
C	845 (56.0)
D	168 (11.1)
X	40 (2.6)
**Reliability**	
Poor	21 (1.4)
Fair	1044 (69.1)
Good	395 (26.2)
Excellent	50 (3.3)

**Table 5:** The most reported potential drug-drug interactions

DDI	Frequency	Risk rating	Description
Paracetamol/Ondansetron	90	B	Ondansetron diminish the analgesic effect of Paracetamol
Cyclophosphamide/Ondansetron	74	B	Ondansetron may decrease the serum concentration of cyclophosphamide
Doxorubicin/Cyclophosphamide	61	C	Cyclophosphamide may enhance the cardiotoxic effect of Anthracyclines
Metformin/Dexamethasone	54	C	Dexamethasone may diminish the therapeutic effect of Metformin
Dexamethasone/Aspirin	44	C	Salicylates may enhance the adverse/toxic effect of corticosteroids. These specifically include GI ulceration and bleeding. Corticosteroids may decrease the serum concentration of salicylates. Withdrawal of corticosteroids may result in salicylate toxicity

### Description of drug-herb and drug-food interactions

The patients have used 0–24 herbs and the mean ± SD was 1.3 ± 1.9. Anise (*Pimpinella anisum L.*) 28.4%, mixed herb 17.1%, Chamomile (*Matricaria chamomilla* L.) 11%, and Sage (*Salvia officinalis L.*) 9.1% were the most used herbs. Only 24 drug-herb interactions were found and 219 drug-miscellaneous product interactions. The most common interactions were Dexamethasone/Grapefruit, Prednisolone/Grapefruit, Paclitaxel/Grapefruit, Atorvastatin/Grapefruit, and Amlodipine/Grapefruit (Table 6). However, the combination that should be avoided ‘X’ was Aprepitant/Grapefruit (*n* = 8).

**Table 6:** The most reported potential drug-herb and drug-food interactions

Drug-herb or drug-food interactions	Frequency	Risk rating	Description
Dexamethasone/Grapefruit	78	C	Grapefruit may increase the serum concentration of Dexamethasone
Prednisolone/Grapefruit	14	B	Grapefruit may increase the serum concentration of prednisolone
Paclitaxel/Grapefruit	13	C	Grapefruit may increase the serum concentration of paclitaxel
Atorvastatin/Grapefruit	11	D	Grapefruit may increase the serum concentration of atorvastatin
Amlodipine/Grapefruit	11	B	Grapefruit may increase the serum concentration of amlodipine

### Univariate and multivariate analysis of potential DDIs

Univariate analysis showed a significant association between the number of DDIs and the following variables: age (*p* < 0.001), duration of treatment (*p* = 0.001), number of comorbid diseases (*p* < 0.001), number of all drugs used (*p* < 0.001), number of anticancer drugs (*p* = 0.001). However, the type and status of cancer were not significantly associated with the number of potential DDIs (Table 7). Furthermore, the Spearman's correlation coefficient showed a significant and positive correlation between the number of observed DDIs and the number of reported symptoms (*p* = 0.032); (Table 8).

**Table 7:** Univariate analysis of potential DDIs

Characteristic	Frequency (%)	No. of DDIs Median [Q1-Q3]	*P*-value
**Age**			** < 0.001** ^a^
≤ 50	127(38.8%)	3.0 (1.0–5.0)	
> 50	200 (61.2%)	4.0 (2.0–8.0)	
**Gender**			0.792 ^a^
Male	103(31.5%)	3.0 (2.0–7.0)	
Female	224 (68.5%)	3.0 (1.0–7.0)	
**BMI**			0.242 ^b^
< 18.5	7 (2.1%)	2.0 (2.0–4.0)	
18.5–24.9	83 (25.4%)	3.0 (1.0–6.0)	
25–29.9	119 (36.4%)	4.0 (2.0–7.0)	
≥ 30	118 (36.1%)	4.0 (2.0–7.0)	
**Cancer type**			0.389 ^a^
Hematological	83 (25.4%)	3.0 (2.0–5.0)	
Solid	244 (74.6%)	4.0 (1.0–7.0)	
**Metastasis**			0.304 ^a^
No	195 (59.6%)	4.0 (2.0–7.0)	
Yes	132 (40.4%)	3.0 (1.0–7.0)	
**Palliative**			0.106 ^a^
No	200 (61.2%)	4.0(2.0–7.0)	
Yes	127 (38.8%)	3.0 (1.0–6.0)	
**Duration of treatment**			**0.001** ^b^
< 1	193 (59.0%)	4.0 (2.0–7.0)	
1–3	90 (27.5%)	3.0 (1.0–6.0)	
> 3	44(13.5%)	2.0 (1.0–4.8)	
**Number of comorbid diseases**			** < 0.001** ^b^
0	165 (50.5)	1.0 (1.0–2.0)	
1	91 (27.8)	2.0 (1.0–4.0)	
≥ 2	71 (21.7)	4.0 (3.0–4.0)	
**All drugs used**			** < 0.001** ^a^
≤ 7	158 (48.3%)	2.0 (1.0–3.0)	
> 7	169(51.7%)	5.0 (3.0–10.0)	
**Anticancer treatment**			**0.001** ^a^
≤ 2	200 (61.2%)	3.0 (1.0–5.8)	
> 2	127 (38.8%)	5.0 (2.0–8.0)	
**Education**			0.314 ^b^
Not educated	18 (5.5%)	6.0 (2.5–9.0)	
Standard < 5	32 (9.8%)	3.0 (0.0–7.0)	
Standard 5–10	105(32.1%)	3.0 (2.0–6.5)	
Standard 11–12	100 (30.6%)	3.0 (1.0–7.0)	
University qualification	72 (22.0%)	3.0 (1.0–6.0)	

^a^The statistical significance of differences was calculated using the Mann–Whitney U test

^b^The statistical significance of the differences was calculated using the Kruskal–Wallis test

The bold *P*-value indicates significant differences

**Table 8:** Spearman’s correlation between the number of reported symptoms and the number of observed DDIs

		**Number of reported symptoms**
**No. of DDIs**	**Correlation Coefficient**	0.118^a^
	***P*-value**	**0.032**

^a^Correlation is significant at the 0.05 level (2-tailed)

Multiple linear regression analyses revealed that the number of potential DDIs decreased significantly with the treatment duration (*p* = 0.007). At the same time, it increased with the number of comorbidities (*p* < 0.001) and the number of drugs used (*p* < 0.001). No multicollinearity was found between the independent variables (Tables 9 and 10).

**Table 9:** Multivariate regression analysis of potential DDIs

	**Model**	**Unstandardized Coefficients**		**Standardized Coefficients**	**t**	*p*-value*	**95.0% Confidence Interval for B**		**Collinearity Statistics**
		B	Std. Error	Beta			Lower Bound	Upper Bound	VIF
**1**	**(Constant)**	.701	.546		1.283	0.200			
	**Age**	.803	.421	.090	1.907	0.057	.237	.106	.081
	**Duration**	-.712	.260	-.118	-2.737	**0.007**	-.157	-.151	-.117
	**Comorbidities**	1.672	.280	.309	5.971	** < 0.001**	.489	.317	.255
	**All drugs used**	3.320	.431	.383	7.710	** < 0.001**	.545	.396	.329
	**Anticancer treatment**	.449	.411	.051	1.094	0.275	.130	.061	.047
	**Number of reported symptoms**	.107	.054	.086	1.967	0.050	.106	.109	.084

The bold *P*-value indicates significant differences

**Table 10:** The quality of multivariable regression model

Model	R	R Square	Adjusted R Square	Std. Error of the Estimate	Change Statistics					Durbin-Watson
					**R Square Change**	**F Change**	**df1**	**df2**	**Sig. F Change**	
1	.645^a^	.416	.405	3.34520	.416	37.924	6	320	.000	1.906

^a^Predictors: (Constant), Age, Duration, Comorbidities, All drugs used, Anticancer treatment, Number of reported symptoms

^b^Dependent Variable: Number of DDIs

## Discussion

Drug-drug interactions (DDIs) are a series of events or circumstances during pharmacotherapy that may cause the patient's expected therapeutic outcomes to be compromised. This study highlights the prevalence and risk rating classification of drug-drug, drug-herb, and drug-food interactions and determines the predictors of DDIs in oncology patients.

In the current study, 68.5% of our sample was female. Female patients accounted for 4,668 cases in Palestine, accounting for more than half (54%) of all cases [2]. Furthermore, solid cancer represented 74.6% of the sample. Similarly, solid tumors (84.8%) account for most cancer cases in Palestine, whereas hematologic malignancies account for 15.2% [2]. Furthermore, breast cancer followed by colon cancer represents the most frequent types in both the study sample and the Palestinian cancer population [2].

According to the findings of this study, the prevalence of DDIs among cancer patients was potentially high (88.1%), which means that the majority of cancer patients had at least one DDI. The percentage is higher compared to other studies, 78% [7] and 24.2% [26]. However, India reported that 88.9% had at least one DDI [27], and in Pakistan, it was 92% [28], and the findings in both studies are similar to our analysis. In a study conducted in Korea specifically on oral antineoplastic agents, the prevalence was highest among the targeted agents (63.2%) followed by traditional agents (21.2%) and endocrine agents (19.3%) [29]. Furthermore, previous studies in Palestine also reported potentially high percentages of DDIs among different populations, such as hemodialysis patients (89.1%) [18] and cardiovascular patients (94%); [19]. However, another study reported a lower percentage (56%) among Palestinian surgical patients [20]. It can be clearly seen that the prevalence of DDIs among the cancer population is extremely high, as described in numerous studies around the world. This is in part due to the use of multiple medications, which requires the development of a clinical oncology pharmacy service to provide pharmacotherapy consultations to prevent any serious DDI, medication errors, and adverse effects related to anticancer medications [30].

Of all 1510 potential DDIs, 857 (56.8%) were classified as moderate interactions, which require medication monitoring. While the majority of the DDIs (56%) had a C category in the risk rating classification and only 11.1% of DDIs that necessitate modifying the medication are in category D. A suitable monitoring strategy should be created to identify the detrimental impacts of Category C interactions. In a small percentage of individuals, dosage changes for one or both medicines may be required. However, in category D, a patient-specific evaluation is required and specific steps must be taken accordingly. Aggressive monitoring, empiric dose modifications, or the use of alternative drugs are examples of these actions.

In our study, patients had 40 interactions (2.6%) with category X. Compared to other studies, this percentage is considered high [7]. However, it was lower than a study conducted in India [27]. The adverse effect of these interactions exceeds the benefits. For instance, Promethazine/Metoclopramide is one of the interactions to be avoided; the probable mechanism for this interaction has been reported as QTc interval prolongation. On the other hand, aprepitant acts as a liver enzyme (cytochrome P450 3A4) inhibitor that affects the metabolism of some cancer drugs and leads to an increase in blood concentration, causing adverse effects related to the drug used, i.e. Doxorubicin/Aprepitant.

Paracetamol/Ondansetron, Cyclophosphamide/Ondansetron, Doxorubicin/Cyclophosphamide, Metformin/Dexamethasone, and Dexamethasone/Aspirin were the most common DDI pairs. Interactions between Cyclophosphamide/Ondansetron and Cyclophosphamide/Doxorubicin were also found in a previous study [7]. Cyclophosphamide may enhance the cardiotoxicity of anthracyclines. However, the interactions, Cyclophosphamide/Ondansetron and Metformin/Dexamethasone, were among the most common in India [27]. The proposed mechanisms of these interactions are that ondansetron may decrease the serum concentration of cyclophosphamide, and dexamethasone may decrease the therapeutic effect of metformin.

In general, the prevalence of complementary and alternative medicine use especially herbal medicine among cancer patients is significantly high in Palestine, accounting for 69.5% [31], and 68% [32] in the two study cohorts, and other studies reported that in different populations in Palestine [33, 34]. Along with traditional therapy, cancer patients, particularly in Middle Eastern nations, also utilize herbal medications more frequently than the general population. The rationale for this use includes lowering the likelihood of recurrence, increasing health status, and reducing the adverse effects of cancer treatment. Furthermore, a lack of awareness and education on drug-herb interactions among physicians, patients, and the general public and research limitations in this area has resulted in several potential negative effects, including direct toxic effects and potential interactions with anticancer drugs [35]. In this study, the most commonly used herbs in cancer patients were anise, mixed herbs, chamomile, and sage. These findings differ from a couple of studies that found ephedra is the most widely used herb in Palestinian cancer patients [31, 32]. Ephedra use was 0.0% in 2011 [31], 55.2% in 2014 [31], 31.1% in 2016 [32], while it was only 3.4% in our study. During 2014 and 2016, the media played a role in promoting the benefit of ephedra in cancer patients [31, 32], which explained the high prevalence of ephedra use at that time, while currently, its decreased use may be due to improved awareness in this population. Furthermore, in terms of the drug-food relationship, a case report described the significant interaction between docetaxel and grapefruit juice by limiting its clearance by more than 15% [36]. One of the most common interactions in our study was Paclitaxel/Grapefruit, while Aprepitant/Grapefruit should be avoided.

The main outcomes of the current study are the large number of observed DIs and the correlation between the number of reported symptoms and the number of DDIs. In fact, this relationship is quite controversial, as the number of reported symptoms could be a potential cause of DIs only if the drugs involved were prescribed as part of a prescribing cascade to alleviate new symptoms of potential adverse events. However, as expected, the number of potential DDIs, the duration of treatment, and the number of comorbidities were all significant in multiple linear regression. In a study conducted in Pakistan in which hospitalized patients, combination chemotherapy, and patients with solid malignancy were all significant in univariate analysis, but they were insignificant in multiple linear regression. The only predictors were the number of all prescribed drugs and anticancer medications [7]. Furthermore, the number of drugs used was significant, which is consistent with the findings of a study conducted in Iran [37]. Other studies have found that as the number of drugs and health problems increases, the risk of DDIs increases in patients receiving antineoplastic therapy [27, 30, 38]. It was reported that one of the consequences of polypharmacy is DDIs, since the patient, especially the elderly, will have several medications to take, multiple health problems, inpatient admissions, and other medications to treat adverse reactions [27]. However, a study conducted by Riechelmann et al. showed that type of cancer and medication indications (to treat comorbid conditions versus supportive care) were associated with DDIs in patients with cancer [12]. In terms of duration of treatment, it was inversely proportioned with the number of DDIs in multiple regression analysis, this result may be attributed to the good knowledge that the patient acquires during the treatment path. We also found a significant and positive correlation between the number of DDIs and the number of reported symptoms. In fact, a more extensive evaluation is necessary regarding this point, as the consequences of DDIs may cause new symptoms or cancer patients may tend to use medications to alleviate their symptoms, thus increasing the risk of DDIs.

### Strengths and limitations

This is the first study in Palestine that describes the prevalence and features of the potential DIs among oncology patients. The main strength of this study is the inclusion of patients with all types of cancer, those who are taking oral or intravenous anticancer medications, and the assessment of the DIs with other substances (such as herbs and foods) that practitioners sometimes neglect. Nonetheless, our study had limitations. First, it has a relatively small sample size, and only two centers are included. Therefore, the results could not be generalized. Second, we did not check the clinical implications of potential DDIs. Third, one screening tool was applied, and some herbs were not found during the searching process. Importantly, there are other available sources of information on drug interactions that may be used at the same time to increase sensitivity to detect potential interactions. Finally, all types of DDIs were included in the analysis even those of low clinical importance, such as classes A and B.

## Conclusions

As mentioned earlier, the prevalence of potential DDIs among the cancer population was extremely high, which required local institutions to develop a comprehensive protocol to monitor and evaluate the DIs by improving doctor-pharmacist communication and supporting the role of clinical pharmacists. For example, the clinical pharmacist should continuously review the medications profile of cancer patients to accurately detect any interactions. On the other hand, many sources detect the interactions, which may differ significantly with respect to the information posted. Therefore, a group consensus should be reached to use a specific interaction screening tool. Furthermore, healthcare professionals must improve patients’ knowledge of self-medication and improve their awareness regarding DIs. We recommend conducting more studies to examine the relationship between observed DDIs and poor health and economic outcomes.

## Data Availability

Due to privacy, the datasets used and/or analysed during the current study are available from the corresponding author on reasonable request.

## Abbreviations

DIs: Drug interactions
SPSS: Statistical Package for the Social Sciences
DDIs: Drug-drug interactions
OTC: Over-the-counter
BMI: Body mass index
IRB: Institutional Review Boards
ALT: Alanine aminotransferase
AST: Aspartate aminotransferase (AST)
MM: Multiple myeloma
NHL: Non-Hodgkin lymphoma
HL: Hodgkin lymphoma
AML: Acute myeloid leukemia
ALL: Acute lymphoblastic leukemia
CLL: Chronic lymphocytic leukemia
